# Affected astrocytes in the spinal cord of the leukodystrophy vanishing white matter

**DOI:** 10.1002/glia.23289

**Published:** 2017-12-29

**Authors:** Prisca S. Leferink, Nicole Breeuwsma, Marianna Bugiani, Marjo S. van der Knaap, Vivi M. Heine

**Affiliations:** ^1^ Department of Pediatrics/Child Neurology Amsterdam Neuroscience, VU University Medical Center Amsterdam The Netherlands; ^2^ Department of Pathology VU University Medical Center, Amsterdam Neuroscience Amsterdam The Netherlands; ^3^ Department of Functional Genomics Center for Neurogenomics and Cognitive Research, Amsterdam Neuroscience, Vrije Universiteit Amsterdam Amsterdam The Netherlands; ^4^ Department of Complex Trait Genetics Center for Neurogenomics and Cognitive Research, Amsterdam Neuroscience, VU Universiteit Amsterdam The Netherlands

**Keywords:** astrocytes, leukoencephalopathy, neuroglia, neuropathology, spinal cord, vanishing white matter disease

## Abstract

Leukodystrophies are often devastating diseases, presented with progressive clinical signs as spasticity, ataxia and cognitive decline, and lack proper treatment options. New therapy strategies for leukodystrophies mostly focus on oligodendrocyte replacement to rescue lack of myelin in the brain, even though disease pathology also often involves other glial cells and the spinal cord. In this study we investigated spinal cord pathology in a mouse model for Vanishing White Matter disease (VWM) and show that astrocytes in the white matter are severely affected. Astrocyte pathology starts postnatally in the sensory tracts, followed by changes in the astrocytic populations in the motor tracts. Studies in post‐mortem tissue of two VWM patients, a 13‐year‐old boy and a 6‐year‐old girl, confirmed astrocyte abnormalities in the spinal cord. For proper development of new treatment options for VWM and, possibly, other leukodystrophies, future studies should investigate spinal cord involvement.

## 1 INTRODUCTION

White matter disorders (WMDs) have many causes and affect both the brain and spinal cord of the central nervous system (CNS). The peripheral nervous system is variably affected. The genetic WMDs, known as leukodystrophies, include disorders like Canavan disease, Vanishing White Matter (VWM), Pelizaeus‐Merzbacher disease (PMD) and metachromatic leukodystrophy (MLD). Leukodystrophies are rare disorders that typically show progressive involvement of the white matter (WM; Ashrafi & Tavasoli, 2017). Clinical signs of leukodystrophies vary and are dependent on the age of onset; they often include loss of motor function due to spasticity and ataxia. Cognitive decline of variable severity occurs as well. Since many patients with leukodystrophies show progressive decline, there is urgent need for better treatment.

Cell replacement therapy, where populations of healthy (macro‐)glial cells (oligodendrocytes and astrocytes) or their precursor cells are transplanted in the CNS, is a promising treatment strategy for leukodystrophies (Osorio & Goldman, 2016). Several glial transplantations have been performed in the brains of rodent models with WM defects, which in successful cases resulted in myelin formation and increased survival (Izrael et al., 2007; Pouya, Satarian, Kiani, Javan, & Baharvand, 2011; Wang et al., 2013). However, proof‐of‐concept studies in animal models that accurately mimic human leukodystrophies are still lacking. A first clinical trial, where human neural stem cells were transplanted in the frontal white matter of patients with PMD, showed no adverse effects and indications of myelination on MRI in the transplanted regions (Gupta et al., 2012). Remarkably, most transplantation studies have so far focused on the brain, while many leukodystrophies also show spinal cord involvement, such as Alexander disease (Liu et al., 2016; van der Knaap et al., 2006), PMD (Koeppen & Robitaille, 2002), MLD (Toldo, Carollo, Battistella, & Laverda, 2005) and Krabbe disease (Wang, Melberg, Weis, Mansson, & Raininko, 2007). In rodent models of spinal cord injury, glial transplantations in the spinal cord resulted in decreased pathology and functional recovery (Haidet‐Phillips et al., 2014; Li et al., 2015; Nicaise, Mitrecic, Falnikar, & Lepore, 2015). Also a clinical trial, where patients with spinal cord injury were transplanted with human neural stem cells, showed moderate clinical improvements (Shin et al., 2015). These findings suggest that glial transplantations could also repair abnormalities in the spinal cord of leukodystrophies.

The spinal cord is a compact structure with a high density of ascending somatosensory tracts and descending motor tracts. Consequently, spinal cord damage, either by spinal cord injury or WMDs, is readily associated with significant neurological handicap. This is in contrast to WM abnormalities in the brain, which may remain without clinical consequences depending on location and extent. Treatment of both the spinal cord and the brain in leukodystrophy patients may be essential for restoration of proper CNS function. Intravenous injection of neural and mesenchymal stem cells in a rodent model of multiple sclerosis (MS) resulted in increased myelination and reduction of pathology in the spinal cord, together with improved locomotor function, highlighting the importance of spinal cord treatment in WMDs (Mitra, Bindal, Eng Hwa, Chua, & Tan, 2015; Zhang et al., 2016). This implies that in leukodystrophies with spinal cord involvement, prospective cellular replacement therapies should also target the spinal cord to achieve CNS regeneration.

To design treatment options targeting the spinal cord, we need understanding of spinal cord pathology in leukodystrophies. Since leukodystrophies often display a lack of myelin, most therapeutic strategies focus on the treatment of oligodendrocytes, the cell type responsible for producing myelin in the CNS. However, other neural cell types, such as astrocytes, are essential for myelin development, integrity and repair (Kiray, Lindsay, Hosseinzadeh, & Barnett, 2016; Lundgaard, Osorio, Kress, Sanggaard, & Nedergaard, 2014). Furthermore, astrocytes play an important role in leukodystrophies (Dooves, Bugiani, et al., 2016; Dooves, van der Knaap, & Heine, 2016; Gorospe & Maletkovic, 2006; Lanciotti et al., 2013; Rodriguez, 2013; van der Knaap & Heine, 2016). In VWM, astrocytes are suggested to be the primary affected cell type (Bugiani et al., 2011; Dooves, Bugiani, et al., 2016). Even though the brain pathology of VWM patients has been studied in great depth (Bugiani et al., 2011), our insight into spinal cord pathology is limited to MRI studies (Eluvathingal Muttikkal, Montealegre, & Matsumoto, 2017; Meoded, Poretti, Yoshida, & Huisman, 2011; van der Knaap et al., 1998). We therefore studied the spinal cord glial cells in a VWM mouse model and in two VWM patients.

## 2 MATERIALS AND METHODS

### 2.1 Human tissue

The spinal cords of two VWM patients were examined. A cross‐section of cervical spinal cord was collected at autopsy from a 13‐year‐old male VWM patient (VWM343), with two compound heterozygous mutations in the *EIF2B5* gene (c.271A > G/p.Thr91Ala and c.1208C > T/p.Ala403Val). The thoracic‐level spinal cord was collected from a 6‐year‐old female VWM patient (VWM367) with two compound heterozygous mutations in the *EIF2B5* gene (c.338G > A/p.Arg113His and c.1208C > T/p.Ala403Val). As control, the tissue of a 10‐year‐old patient deceased of metastasized osteosarcoma was used. For cryo‐sectioning, the spinal cord of VWM343 was fixed in 4% PFA for 2 days, after which the tissue was embedded in optimal cutting temperature (OCT) mounting solution and stored at −80°C until further processing.

The human post‐mortem spinal cord tissue was collected at the VU University Medical Center in Amsterdam, the Netherlands, with approval by the Institutional Review Board and informed consent of the parents.

### 2.2 Experimental animals

The VWM mice were homozygous for a mutation in the *Eif2b5* gene (*Eif2b5* ^Arg191His/Arg191His^), on a C57Bl/6J background strain (referred to as *2b5^ho^*; Dooves, Bugiani, et al., 2016). Heterozygous littermates, referred to as *2b5^he^*, were used as controls. Mice of embryonic ages E13.5 and E18.5 were harvested, after which they were fixated in 2% paraformaldehyde for 48 hours. The tails were used for genotyping. Adult mouse spinal cords were harvested at 4, 7.5, 8, 9, and 10 months of age, after transcardial perfusion with 4% paraformaldehyde, followed by 48 hr post‐fixation. The spinal cords were embedded in OCT mounting solution (Sakura Finetek Europe, Alphen a/d Rijn, Netherlands) and stored at −80°C until further processing.

Experimental procedures involving mice were in strict compliance with animal welfare policies of the Dutch government and were approved by the IACUC of the VU University, Amsterdam.

### 2.3 (immuno)histochemistry

The paraffin‐embedded thoracic spinal cords of patient VWM367 and control were cut at a thickness of 5 μm, histochemically labelled according to standard procedures with haematoxylin‐eosin (H&E), and immunolabeled with antibodies against nestin (mouse, 1:500, BD Biosciences, Franklin Lakes, New Jersey, U.S. 611658), Olig2 (rabbit, 1:500, Millipore, Billerica, Massachusetts, U.S. AB9610), glial fibrillary acid protein (GFAP; rabbit, 1:1000, DAKO, Glostrup, Denmark Z0334) and proteolipid protein (PLP; mouse, 1:500, Serotec, Bio‐Rad, Hercules, California, U.S. MCA839 G).

H&E stains were performed on the of 12 µm thick cryo‐sections of the mouse tissue. The slides were washed six times for 5 min in phosphate buffered saline (PBS), at room temperature, to wash away the OCT. The sections were briefly incubated in milliQ water, stained with hematoxylin, and rinsed with tap water. The sections were then differentiated in 1% acid alcohol, rinsed again with tap water, incubated in 0.1% sodium carbonate, and rinsed again with tap water. Then sections were stained with Eosin Y, followed by washing steps with tap water and milliQ water. Then the slides were dehydrated with 50%–100% alcohol, cleared in Xylene, and embedded in Depex.

### 2.4 Immunofluorescence

Spinal cords of patient VWM343 and VWM mice were cryo‐sectioned at 12 µm thickness and immunofluorescently labeled. To wash away the cryo‐protectant, the slides were washed six times for 5 min in PBS at room temperature. To increase antibody retrieval, the slices received a microwave pre‐treatment at 90°C in 0.1M citrate buffer pH 6 for 10 min, after which the slides were allowed to cool to room temperature. The slides were washed one time with PBS, followed by a 1‐hr blocking step with blocking buffer (PBS + 5% NGS + 0.1% BSA + 0.3% Triton X‐100) at room temperature. Primary antibodies against nestin (mouse, 1:500, BD Biosciences, Franklin Lakes, New Jersey, U.S. 611658), GFAP (rabbit, 1:1000, DAKO, Glostrup, Denmark Z0334), Olig2 (rabbit, 1:500, gift of J.H. Alberta, Harvard University, Boston, MA), Sox9 (rabbit, 1:500, Cell Signaling, Danvers, Massachusetts, U.S. 82630), Nuclear Factor I‐A (NFIA; rabbit, 1:500, Active Motif, Carlsbad, California, U.S. 39397), Sox2 (rabbit, 1:1000, Millipore, Billerica, Massachusetts, U.S. AB5603), inhibitor of DNA binding 3 (Id3; rabbit, 1:500, Cell Signaling, Danvers, Massachusetts, U.S. 9837), Pax3 (mouse, 1:50, Hybridomabank, Iowa City, Iowa, U.S.), Pax6 (mouse, 1:50, Hybridomabank, Iowa City, Iowa, U.S.), Nkx2.2 (mouse, 1:50, Hybridomabank, Iowa City, Iowa, U.S.) Nkx6.1 (mouse, 1:100, Hybridomabank, Iowa City, Iowa, U.S.), vimentin (mouse, 1:400, Sigma, Saint Louis, Missouri, U.S. V5255), α‐smooth muscle actin (mouse, 1:1000, Progen, Heidelberg, Germany 61001), and cleaved caspase 3 (rabbit, 1:400, Cell signaling, Danvers, Massachusetts, U.S. 9661) were diluted in blocking buffer, incubated for 30 min at room temperature, followed by overnight incubation at 4°C. The next day, the primary antibody was washed away six times for 5 min in PBS at room temperature, and secondary antibodies goat‐anti mouse Alexa Fluor 488 or Alexa Fluor goat‐anti rabbit 594 (1:1000, Molecular Probes, Eugene, Oregon, U.S.) were diluted in blocking buffer, and incubated for 1.5 hr at room temperature. The secondary antibody was washed away six times for 5 min in PBS at room temperature, followed by application of nuclear marker 4',6‐diamidino‐2‐phenylindole staining (DAPI, 1:1000) in PBS for 2 min. The tissue slides were embedded in FluormountG (Southern Biotech, Birmingham, Alabama, U.S.) and cover‐slipped.

### 2.5 Quantification and statistics

A Leica DM6000B microscope (Leica Microsystems) was used to take pictures. To image entire spinal cords (Figure 4), two separate photos were merged in Adobe Photoshop CS6 using the “photo merge” tool. The spinal cords were cut out from the image, using the magnetic lasso tool, and pasted on a black background. For quantification of nuclear markers Sox2, Olig2, Sox9, and Id3, photos were taken of the white matter regions of the thoracic spinal cords. Per animal, 3–6 serial sections were analyzed. Image J software was used to outline the white matter, in which the positive cells were counted and expressed as the number of positive nuclei/mm^2^ tissue. A *t* test of equal means was performed to compare means between mutant and controls. All statistical tests were performed using IBM SPSS Statistics version 22.

## 3 RESULTS

### 3.1 Glial cells in the white matter of the adult VWM mouse spinal cord are abnormal

**Figure 1:**
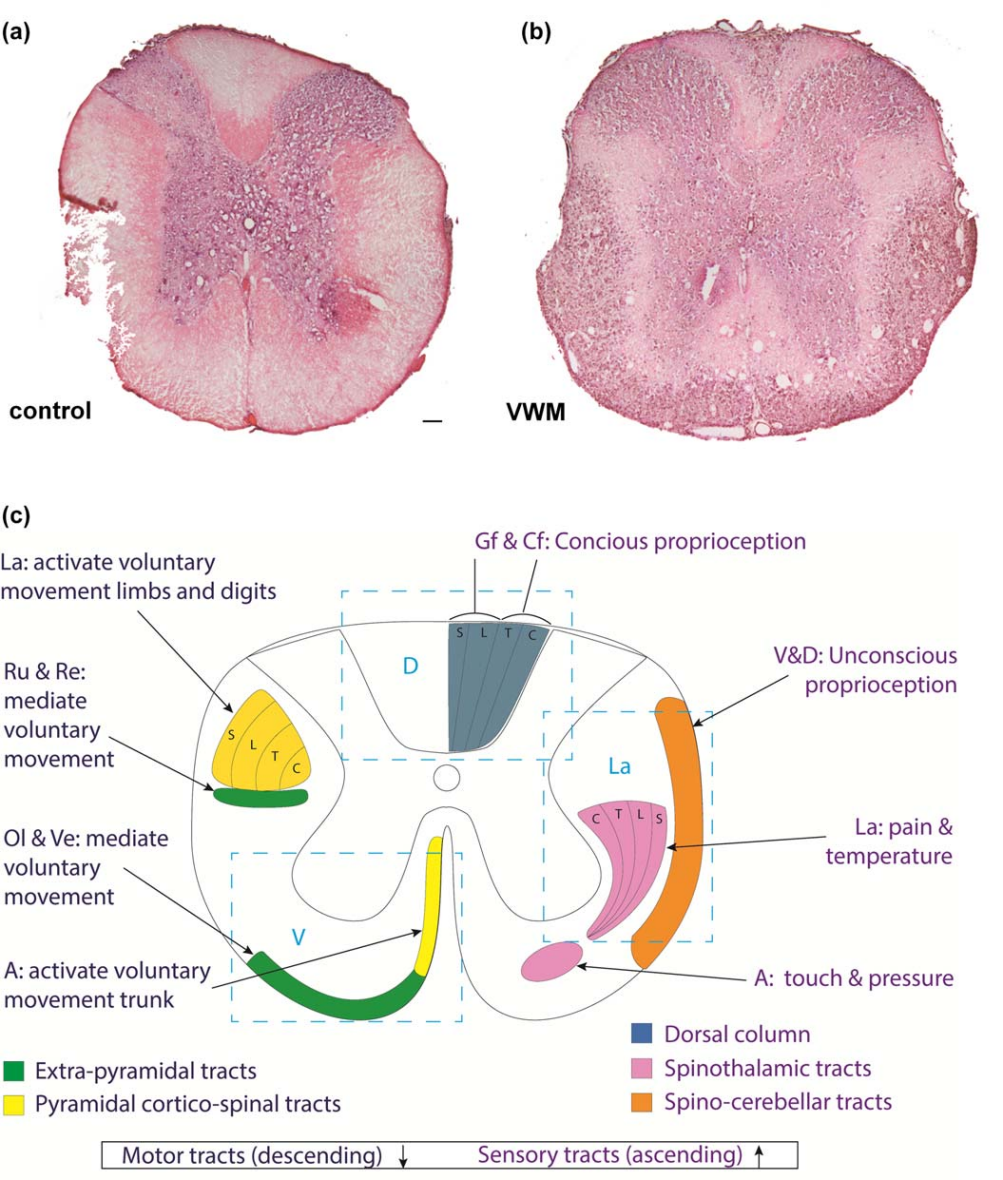
The white matter of the VWM mouse model spinal cord is affected. H&E staining of the thoracic area of the spinal cord of 7.5‐month‐old, (a) *2B5^he^* (control) and (b) *2B5^ho^* (VWM) mouse. Scale bar 75 µm. (c) Schematic representation of white matter tracts in the thoracic adult spinal cord. On the left are the motor tracts depicted, on the right the sensory tracts. In blue rectangular dotted lines are the cut‐outs from the dorsal, lateral and ventral spinal cord regions are depicted as used in the rest of the figures. Gf = Gracile fasciculus, Cf = Cuneate fasciculus, S = sacral, L = lumbar, T = thoracic, C = cervical, V = ventral, D = dorsal, La = lateral, Ru = Rubrospinal tract, Re = Reticulospinal tract, Ol = Oliviospinal tract, Ve = Vestibulospinal tract [Color figure can be viewed at wileyonlinelibrary.com]

To investigate whether the WM of the spinal cord of VWM mice is affected, we performed H&E staining on the spinal cord of 7.5‐month‐old *2b5^ho^* (VWM) and *2b5^he^* (control) mice (Figure 1a,b). The WM tissue was lesioned in all regions of the VWM mouse model spinal cord (Figure 1a,b). The radial organization of the glial cells in the WM was lost. The WM pathology was most severe in the WM directly under the pial surface, in the areas corresponding to the sensory spinocerebellar and spinothalamic tracts (lateral WM) and the gracile fasciculus (dorsal WM), and vestibolospinal motor tracts (ventral WM; Figure 1c). The pathology was less apparent in WM areas adjacent to the gray matter, in the areas corresponding to the cuneate fasciculus (dorsal WM) and corticospinal motor tracts (lateral and ventral WM; Figure 1c). The gray matter appeared unaffected (Figure 1a,b).

**Figure 2:**
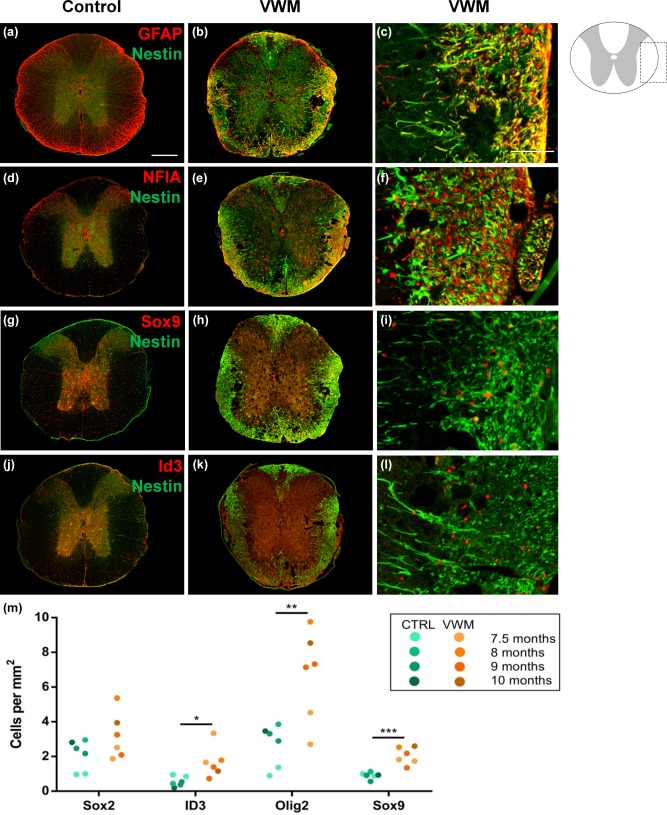
Glial abnormality and cell density in the white matter of the VWM mouse model. Immunocytochemical analysis of the thoracic area of the spinal cord of 7.5‐months‐old mice for nestin in combination with, (a–c) GFAP, (d–f) NFIA, (g–i) Sox9, and (j–l) Id3 in *2B5^he^* (control; a, d, g, j) and *2B5^ho^* (VWM; b, c, e, f, h, i, k, l) mice. a, b, d, e, g, h, j, and k show whole spinal cords, scale bar 250 µm. c, f, i, l show a close up of the lateral white matter area, scale bar 50 µm. (m) Quantification of Sox2‐, Id3‐, Olig2‐, and Sox9‐expressing cells in the WM of the thoracic area of the spinal cord of 7.5‐months‐old mice, expressed as the number of positive cells per mm^2^ [Color figure can be viewed at wileyonlinelibrary.com]

To investigate glial cell pathology, the mouse spinal cords were stained for the astrocyte markers GFAP, Id3, and Sox9, glial marker NFIA, OPC marker Olig2, neural stem cell marker Sox2 and intermediate filament protein marker nestin (Figure 2). The WM regions of the adult VWM spinal cord showed increased expression of nestin, which co‐localized with GFAP (Figure 2b,c), NFIA (Figure 2e–f), Sox9 (Figure 2h,i), and Id3 (Figure 2k,l). No nestin expression was observed in the adult spinal cord of the control mice (Figure 2a,d,g,j). The sub‐pial region of the WM showed increased nestin expression, while WM regions adjacent to the gray matter were relatively spared. The GFAP‐positive cells looked dysmorphic and did not show the radial arrangement seen in the control mice (Figure 2a). NFIA expression was nuclear in controls and in the gray matter of the mutant spinal cords (Figure 2d), while the WM of the mutant spinal cords also showed perinuclear NFIA expression (Figure 2e,f). For this reason, NFIA was not further quantified. Nuclear staining for Sox9 (Figure 2g–i) and Id3 (Figure 2j–l) was observed throughout the spinal cords of both the control and VWM spinal cords. The density of Sox2, Id3, Olig2 and Sox9 in the WM thoracic spinal cord was quantified (Figure 2m). The data points of 7.5‐month‐ (*n* = 2), 8‐month‐ (*n* = 1), 9‐month‐ (*n* = 2) and 10‐month‐ (*n* = 1) old animals were pooled. The density of Id3‐ (*P = *.015), Olig2‐ (*P = *.006) and Sox9‐ (*P = *.001) expressing cells was significantly increased in VWM spinal cords (Figure 2m).

Staining for vimentin (9‐month‐old animals, *n* = 4 for both VWM and control mice) showed co‐localization with GFAP and was therefore considered astrocyte‐specific (Supporting Information, Figure S1b,c). However, vimentin did not specifically label the affected astrocyte population, as it was also expressed in astrocytes outside the affected areas and in the astrocytes of control mice (Supporting Information, Figure S1b,c). The morphology of the blood vessels in the spinal cord was assessed with the marker α smooth muscle actin (α‐SMA; 9‐month‐old animals, *n* = 3 for both VWM and control mice). No abnormalities in the blood vessels were observed (Supporting Information, Figure S2). In the affected WM areas, an increased density of cells could be observed when using DAPI staining (Supporting Information, Figure S1 and 2). As these cells did not show immunoreactivity for any of the tested markers (Sox2, NFIA, Sox9, Olig2, NG2, MBP, SMI, Reelin, Neurofilament, Nestin, Id3, GFAP, S100B, CD68; data not shown) their identity is unknown. To investigate whether these cell populations consist of apoptotic cells, immunohistochemistry was performed for apoptosis marker Cleaved Caspase 3 (CC3) in combination with nestin (Supporting Information, Figure S3; 9‐month‐old animals, *n* = 3 for both VWM and control mice). Even though some CC3‐expressing cells could be observed in the spinal cord (Supporting Information, Figure S3a,b), these were present in both the affected and unaffected areas in the VWM mice, as well as in control mice. The majority of the unidentified cells within the affected areas did not express CC3 (Supporting Information, Figure S3c).

### 3.2 The embryonic VWM mouse spinal cord shows no defects in glial cell development

**Figure 3:**
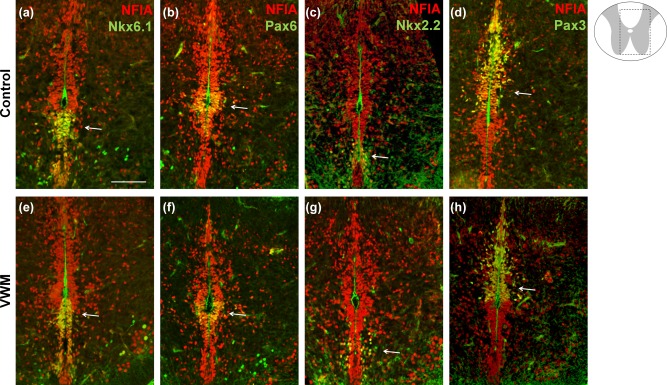
Regional glial patterning during embryonic E 13.5 VWM development is unaffected. Immunocytochemical analysis of the E13.5 spinal cords using glial marker NFIA, in combination with, (a and e) Nkx6.1, (b and f) Pax6, (c and g) Nkx2.2, and (d and h) Pax3 in *2B5^he^* (control; a–d) and in *2B5^ho^* (VWM; e–h) mice. Arrows indicate the area of specific marker expression. Scale bar 75 µm [Color figure can be viewed at wileyonlinelibrary.com]

**Figure 4:**
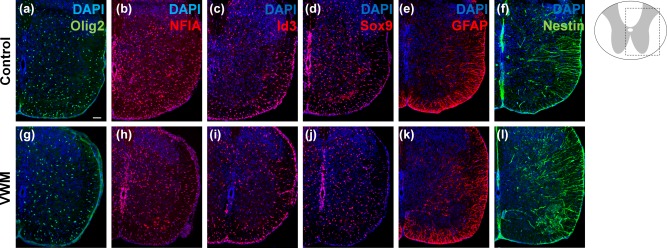
Glial specification in the embryonic E18.5 VWM spinal cord is unaffected. Immunocytochemical analysis of the E18.5 spinal cords using glial markers, (a and g) Olig2, (b and h) NFIA, (c and i) Id3, (d and j) Sox9, (e and k) GFAP, and (f and l) nestin in *2B5^he^* (control; a–f) and in *2B5^ho^* (VWM, g–l) mice. All sections are counterstained with nuclear marker DAPI. Scale bar 75 µm [Color figure can be viewed at wileyonlinelibrary.com]

To study whether glial defects start during early developmental stages, we studied the spinal cord of VWM mice at E13.5 and E18.5. To identify the different neural progenitor populations, which arise during early patterning, we performed immunohistochemical stains for NFIA in combination with regional identity markers Nkx6.1, Pax6, Nkx2.2 and Pax3 (Figure 3). Marker expression was unaffected in VWM mice at E13.5 (Figure 3a–h) and E18.5 (data not shown). Our data further showed that early glial development, assessed with the markers Olig2, NFIA, Id3, Sox9, GFAP and nestin, was unaffected in the VWM mouse spinal cords at E18.5 (Figure 4a–l). Also at E13.5, no abnormalities in Olig2, Sox9, Id3, Sox2 and nestin were observed (Supporting Information, Figure S4)

### 3.3 VWM spinal cord pathology starts at dorsal and lateral white matter

**Figure 5:**
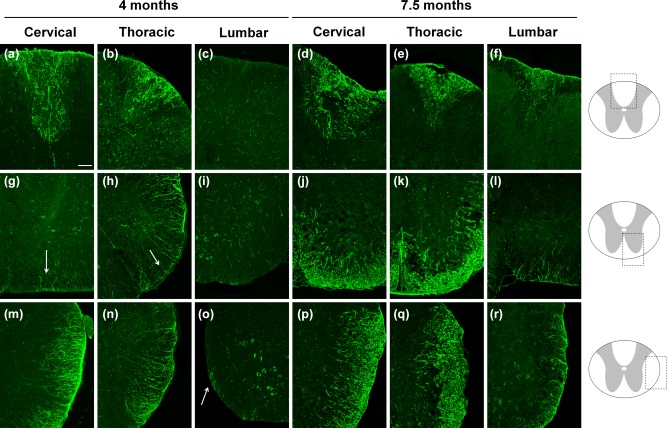
Nestin pathology originates in the lateral and dorsal white matter of the thoracic level. Immunocytochemical analysis for nestin in the spinal cord of (a–c, g–i, m–o) 4‐months‐old and (d–f, j–l, and p–r) 7.5‐month‐old *2B5^ho^* VWM mice at the cervical (a, d, g, j, m, and p), thoracic (b, e, h, k, n, and q), and lumbar (c, f, i, l, o, and r) levels of the dorsal (a–f), ventral (g–l), and lateral (m–r) white matter areas. Arrows indicate areas of starting nestin expression. Scale bar 75 µm [Color figure can be viewed at wileyonlinelibrary.com]

To investigate whether glial populations are affected in specific regions of the WM, we performed immunohistochemical labeling for glial and stem cell markers on different cross sections at the cervical, thorax and lumbar level of the spinal cord of 4‐ and 7.5‐month‐old VWM and control mice (Figure 5). At 4 months, the dorsal and the lateral WM of the cervical and thoracic levels, showed strong nestin expression (Figure 5a,b,m,n). The nestin expression in the ventral thoracic WM was less prominent (Figure 5h), and minimal at the cervical level (Figure 5g). The lumbar level only showed little nestin expression in the lateral WM (Figure 5o); the dorsal and ventral WM regions were unaffected (Figure 5c,i). At 7.5 months, all the WM areas, at all the spinal cord levels inspected, showed nestin expression (Figure 5d–f, j–l,p–r). However, the pathology was, again, least strong at the lumbar level (Figure 5f,l,r), especially in the ventral WM (Figure 5l)

### 3.4 Glial cells in the human VWM spinal cord are severely affected

**Figure 6:**
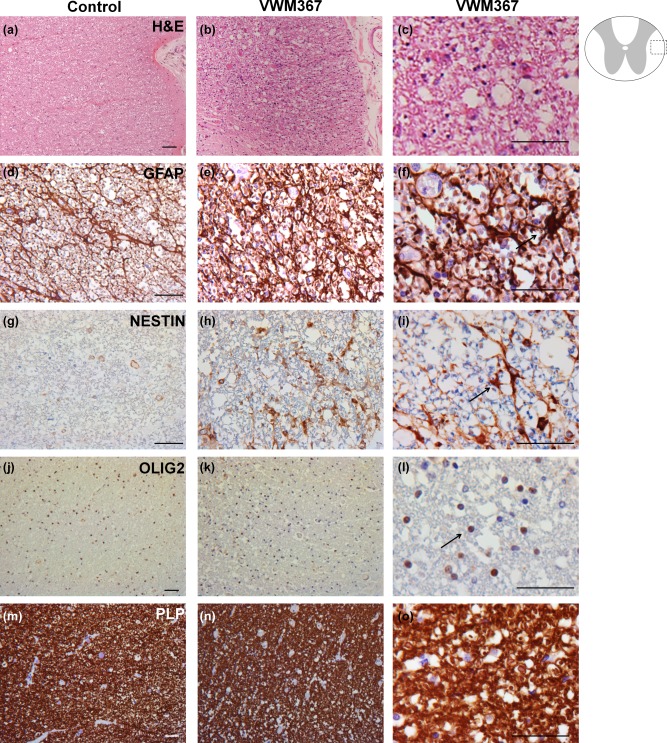
The glial cells in the spinal cord white matter of a VWM patient are affected. Stains on a cross‐section of lateral white matter of the thoracic spinal cord of (a, d, g, j, and m) a healthy control, and (b, c, e, f, h, i, k, l, n, and o) VWM patient VWM367. Stains are performed for (a–c) H&E; (d–f) GFAP; (g–i) nestin; (j–l) Olig2; (m–o) PLP. Arrows indicate examples of (f) a dysmorphic astrocyte; (i) a nestin‐expressing cell; (o) an Olig2‐positive nucleus. Scale bar 75 µm [Color figure can be viewed at wileyonlinelibrary.com]

To study whether human VWM patients show spinal cord abnormalities, we performed microscopic analysis of two VWM patients (Figure 6). H&E staining of the lateral white matter in the thoracic spinal cord of patient VWM367 (Figure 6b,c) showed increased cellular density, compared with control, and vacuolization of the WM (Figure 6a). Since glial cells in the brain WM of VWM patients are abnormal (Bugiani et al., 2011), we studied the astrocytes and oligodendrocytes in the spinal cord of patient VWM367. GFAP‐positive astrocytes in the patient WM displayed an abnormal morphology (Figure 6e, f) compared with the control spinal cord (Figure 6d), with short blunt processes (Figure 6e, f). The gray matter astrocytes were normal. Furthermore, while cells expressing immature intermediate filament protein nestin were absent in the control spinal cord (Figure 6g), nestin‐expressing cells with astrocytic morphology were abundant in the VWM patient spinal cord (Figure 6h,i). The density of cells expressing oligodendrocyte precursor cell (OPC) marker Olig2 was increased in the patient (Figure 6k,l) compared with control tissue (Figure 6j). Staining for myelin marker PLP showed vacuolization in the patient (Figure 6n,o) compared with control (Figure 6m). However, the myelin sheaths looked normal in the VWM patient.

**Figure 7:**
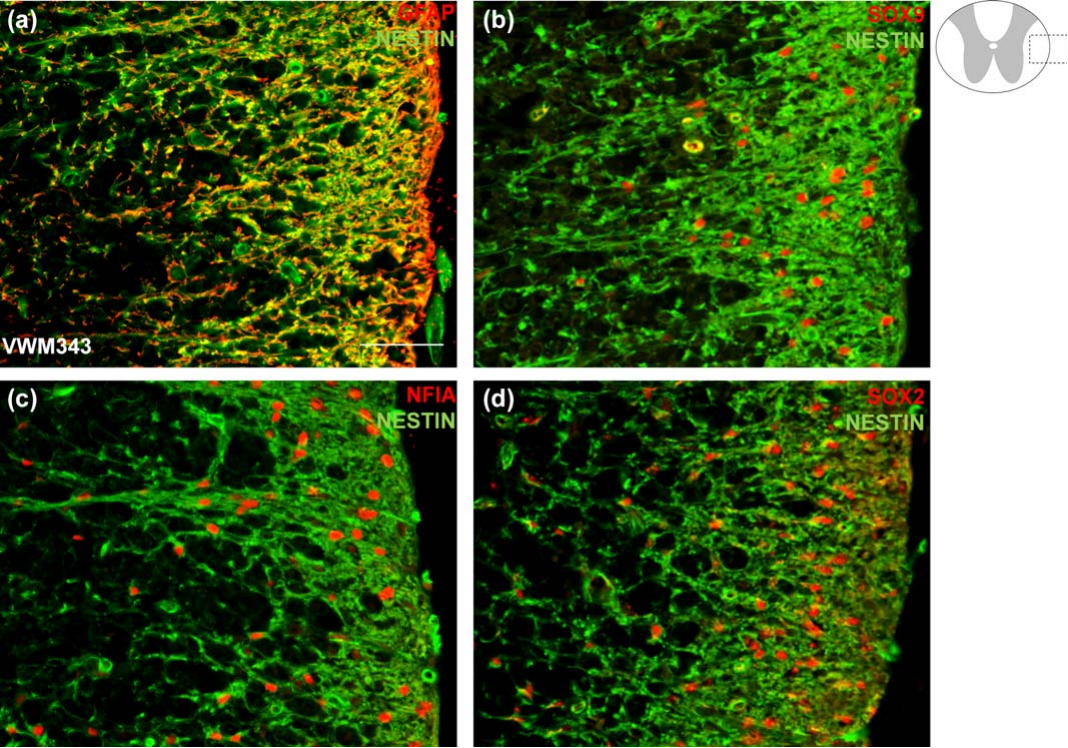
The nestin‐expressing cells in the spinal cord white matter of a VWM patient are astrocytes. A cross‐section of lateral white matter of the cervical spinal cord of VWM patient VWM343 was immunocytochemically analyzed for nestin in combination with, (a) GFAP, (b) SOX9, (c) NFIA, and (d) SOX2. Scale bar 50 µm [Color figure can be viewed at wileyonlinelibrary.com]

To further investigate the nature of the nestin‐expressing cells, immunofluorescent stains were performed on the cervical spinal cord of patient VWM343 for glial and astrocyte marker NFIA, the astrocyte markers GFAP and SOX9, and neural stem cell marker SOX2, in combination with nestin (Figure 7a–d). We showed that the nestin‐expressing cells in the WM co‐localized with GFAP, NFIA, SOX9 and SOX2 (Figure 7a–d), indicating that the nestin‐positive cells are abnormal astrocytes. Unlike nestin, vimentin labeled all astrocytes, and not only the abnormal astrocytes in the spinal cord of patient VWM363 (Supporting Information, Figure S1a). However, vimentin also visualized the dysmorphic morphology of the affected astrocytes, indicating blunt and short processes (Supporting Information, Figure S1a).

## 4 DISCUSSION

While many leukodystrophies have spinal cord involvement, detailed investigation of the pathology is often lacking. Although spinal cord pathology may significantly contribute to neurological disease, the spinal cord is often overlooked as potential therapeutic target. In this study we investigated glial pathology in post‐mortem spinal cord tissue of a VWM mouse model and 2 VWM patients. We showed that the glial cells in the spinal cord of a representative mouse model for VWM (Dooves, Bugiani, et al., 2016) are severely affected. The cellular density in the WM is increased, dysmorphic, nestin‐expressing astrocytes are abundantly present, OPCs are increased in density, and lesions are present throughout the WM. The spinal cord of VWM patients is the same as the mouse spinal cord pathology and confirms astrocyte pathology in WM of the spinal cord. Altogether, our findings show spinal cord pathology in VWM with predominant astrocyte involvement, which has implications for therapy development. Our findings also suggest that spinal cord pathology in other leukodystrophies should receive proper attention.

Our data reveals that astrocytes are most prominently affected in the WM of the VWM spinal cord, which is in line with earlier studies showing that astrocytes are central in the brain pathophysiology of VWM (Bugiani et al., 2011; Dooves, Bugiani, et al., 2016). We report that pathology starts in the dorsal and lateral WM tracts, with most severe defects in the sensory tracts, followed later by the vestibulospinal motor tracts. The pyramidal motor tracts appear unaffected. We further show that the pathology starts at the thoracic level, followed by the cervical level and, later, also by the lumbar level. Recent studies indicate that astrocytes are regionally, morphologically and functionally heterogeneous (Bayraktar, Fuentealba, Alvarez‐Buylla, & Rowitch, 2014; Chaboub & Deneen, 2012; Molofsky & Deneen, 2015; Schitine, Nogaroli, Costa, & Hedin‐Pereira, 2015; Yoon, Walters, Paulsen, & Scarisbrick, 2017). Subpopulations might be selectively affected and might correlate with variation in WM pathology. Indeed, the brains of various leukodystrophies show variation in vulnerability, with for instance sparing of the U‐fibres underneath the cortex (van der Knaap et al., 1997; van Rappard, Boelens, & Wolf, 2015). Microenvironmental factors may contribute to this heterogeneity in pathology, such as regions with neuronal subpopulations with distinct axonal signals, distribution of the vasculature bed, timing of myelination programs, and presence of other neural cells. Therefore, while many environment‐derived factors that regulate myelination processes have been described (Leferink & Heine, 2018), regulatory processes can be specific for particular brain and/or spinal cord regions and thus determine local pathology.

During embryonic development of the spinal cord, regionally distinct subtypes of astrocytes can be identified, which keep their regional location into adulthood. Gradients of morphogens induce patterning of distinct progenitor domains (Briscoe, Pierani, Jessell, & Ericson, 2000; Muhr, Andersson, Persson, Jessell, & Ericson, 2001), which give rise to neurons and glial cells (Pringle, Guthrie, Lumsden, & Richardson, 1998; Rowitch, 2004; Tsai et al., 2012; Wilson & Maden, 2005). Several of these glial (progenitor) populations can be identified by expression of the transcription factors Id3, Sox9, Olig2, and NFIA. Previous studies in rodents showed that Id3 is only expressed in developing astrocytes (Lamantia, Tremblay, & Majewska, 2014; Molofsky et al., 2013). Sox9 is initially (E8.5–E18.5) expressed in all glial (precursor) cells, and is subsequently downregulated in myelinating oligodendrocytes (Stolt et al., 2003) and becomes astrocyte‐specific (Sun et al., 2017). Also NFIA is initially (E11.5) expressed in all developing glial cells, but downregulates Olig2 and becomes exclusive to astrocytes at E13.5–E16.5 (Deneen et al., 2006; Molofsky et al., 2012). In the developing neural tube of rats, neural stem cell marker nestin is expressed directly after closing of the neural tube at E11 (Lendahl, Zimmerman, & McKay, 1990), whereas GFAP expression starts later at E18 (Oudega & Marani, 1991). Vimentin is expressed in the rodent spinal cord from E11 onwards (Oudega & Marani, 1991; Schnitzer, Franke, & Schachner, 1981). Three distinct subtypes of WM astrocytes can be discriminated in the developing spinal cord: Pax6 and Reelin‐expressing astrocytes in p1 domain (VA1); Pax6‐, Reelin‐, Nkx6.1‐, and Slit1‐expressing astrocytes in ventral p2 domain (VA2); and Nkx6.1‐ and Slit1‐expressing astrocytes in p3 domain (VA3; Hochstim, Deneen, Lukaszewicz, Zhou, & Anderson, 2008). Olig2 is expressed in the ventral spinal cord motor neuron domain (pMn), and gives rise to motor neurons and oligodendrocytes. Furthermore, Nkx2.2 marks ventral regions, while Pax3‐expressing cells are located in the dorsal domain. While our results show that embryonic neural tube patterning and glial specification are unaffected in VWM, we found that, in adulthood, the dorsal and lateral WM astrocytes are affected first, followed by ventral WM astrocytes. Although adult astrocytes no longer express the embryonic regional markers, earlier studies showed that the regional identity of astrocytes remains unchanged after transplantation, aging, or injury (Krencik, Weick, Liu, Zhang, & Zhang, 2011; Tsai et al., 2012). The location of the affected astrocyte subpopulation can therefore already indicate the embryonically pattered subtype. New RNA sequencing methods, such as single cell sequencing (Marques et al., 2016) and fluorescence *in situ* sequencing of RNA (FISSEQ; (Lee et al., 2015)), will soon provide more insight into the glial subtypes and hopefully insight into the basis of selective vulnerability in VWM patients.

To start autologous glial cell transplantation in the affected CNS regions, including the spinal cord, there are still issues to overcome. Firstly, we need to clarify which type of cells requires replacement. One strategy would be the transplantation of neural stem cells. While these cells have high proliferative capacities and migration potentials, the differentiation of multipotent stem cells upon transplantation is hard to control; they can potentially generate undesired neural cell types. As astrocytes appear most affected and the myelin sheaths look normal, the transplantation of immature astrocytes would be a promising option. However, the cellular density of Olig2‐positive OPCs is increased, similar to in the brain (Bugiani et al., 2011). This indicates an increased number of immature premyelinating OPCs, and therefore primary oligodendrocyte pathology cannot be excluded (Bugiani et al., 2011). As the human induced pluripotent stem cell (iPSC) technology is increasingly advancing, disease‐modelling studies could identify vulnerable glial cell types in VWM (Nevin et al., 2017). Secondly, the generation of transplantable cells that are safe and are not rejected by the patient is challenging. Autologous transplantation of patients’ own genetically corrected iPSC‐derived astrocytes could be a promising treatment strategy for leukodystrophies (Osorio & Goldman, 2016). Thirdly, as the spinal cord is a highly compact and structured part of the CNS, cellular injection forms a challenge for transplantation. Administration of cell transplants via the cerebral spinal fluid (CSF) could be considered (Liu & Huang, 2008). As we demonstrated that the most affected WM of the spinal cord is located under the pial surface, directly adjacent to the CSF, limited cellular integration could already target the affected regions. Finally, the involvement of the (diseased) microenvironment needs attention (Dooves, van der Knaap, et al., 2016; Leferink & Heine, 2018). Transplanted astrocytes can integrate and become functional (Zhang et al., 2016), but depend on the host microenvironment to give functional recovery in the injured spinal cord (Noble, Davies, Mayer‐Proschel, Proschel, & Davies, 2011). Therefore, the transplantation of healthy glial cells might not be sufficient in VWM, and possibly in many other leukodystrophies. Instead, a combined treatment of astrocyte transplantation, together with modulating interventions to make the affected microenvironment more permissive, could increase the success of the therapy in leukodystrophies (Dooves, van der Knaap, et al., 2016).

In conclusion, our findings show selective astrocyte pathology in the VWM spinal cord. Many other leukodystrophies also have spinal cord involvement, but detailed investigation of spinal cord pathology is often lacking. For proper development of new treatment options, future basic and clinical studies should include investment of spinal cord pathology. As the first clinical trial of neural stem cell transplantation in the brains of PMD patients showed promising safety outcomes (Gupta et al., 2012), future studies should consider also targeting the spinal cord.

## CONFLICT OF INTEREST STATEMENT

The authors declare that they have no conflict of interest.

## Supporting information

Additional Supporting Information may be found online in the supporting information tab for this article.

Supplementary Figure 1

Supplementary Figure 2

Supplementary Figure 3

Supplementary Figure 4

Supplementary Figure Legends
