# Supplementary figures and images for: Affected astrocytes in the spinal cord of the leukodystrophy vanishing white matter
Source: Glia. 2017 Dec 29;66(4):862–73. doi: 10.1002/glia.23289 (PMC5838785; doi:10.1002/glia.23289)

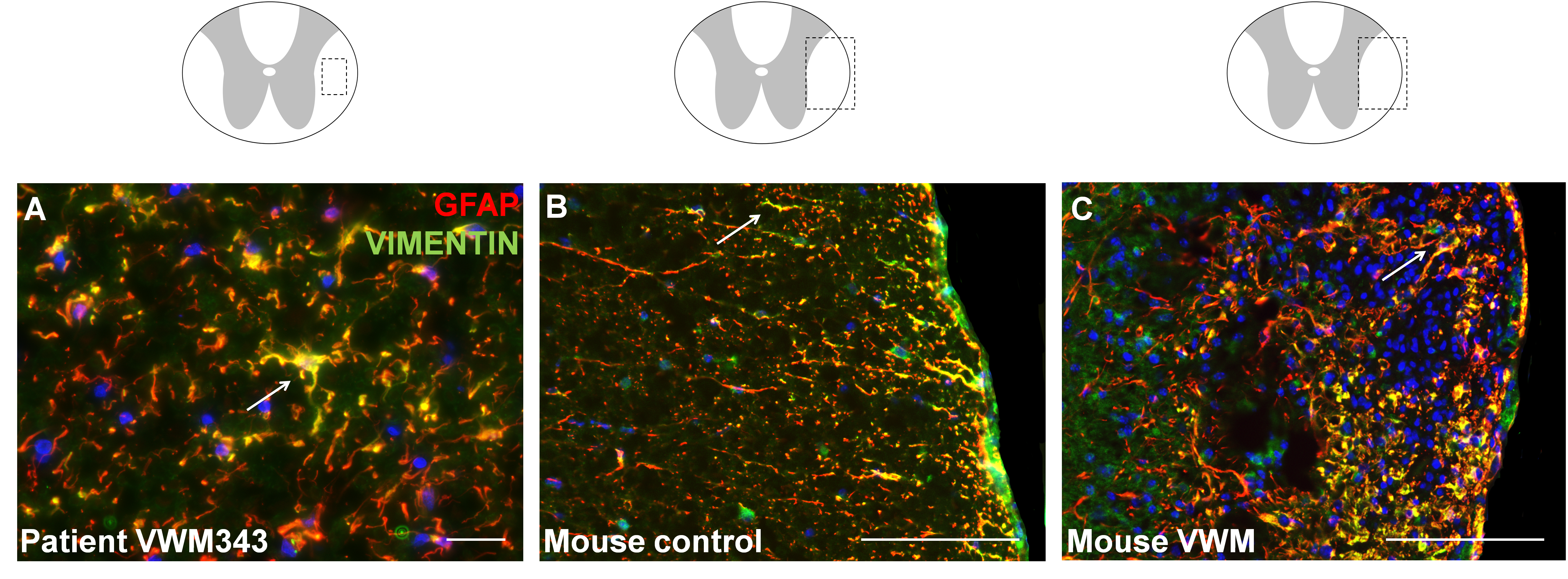

Supplement: Supplementary file 1 — Supplementary Figure 1 [file GLIA-66-862-s001.tif]

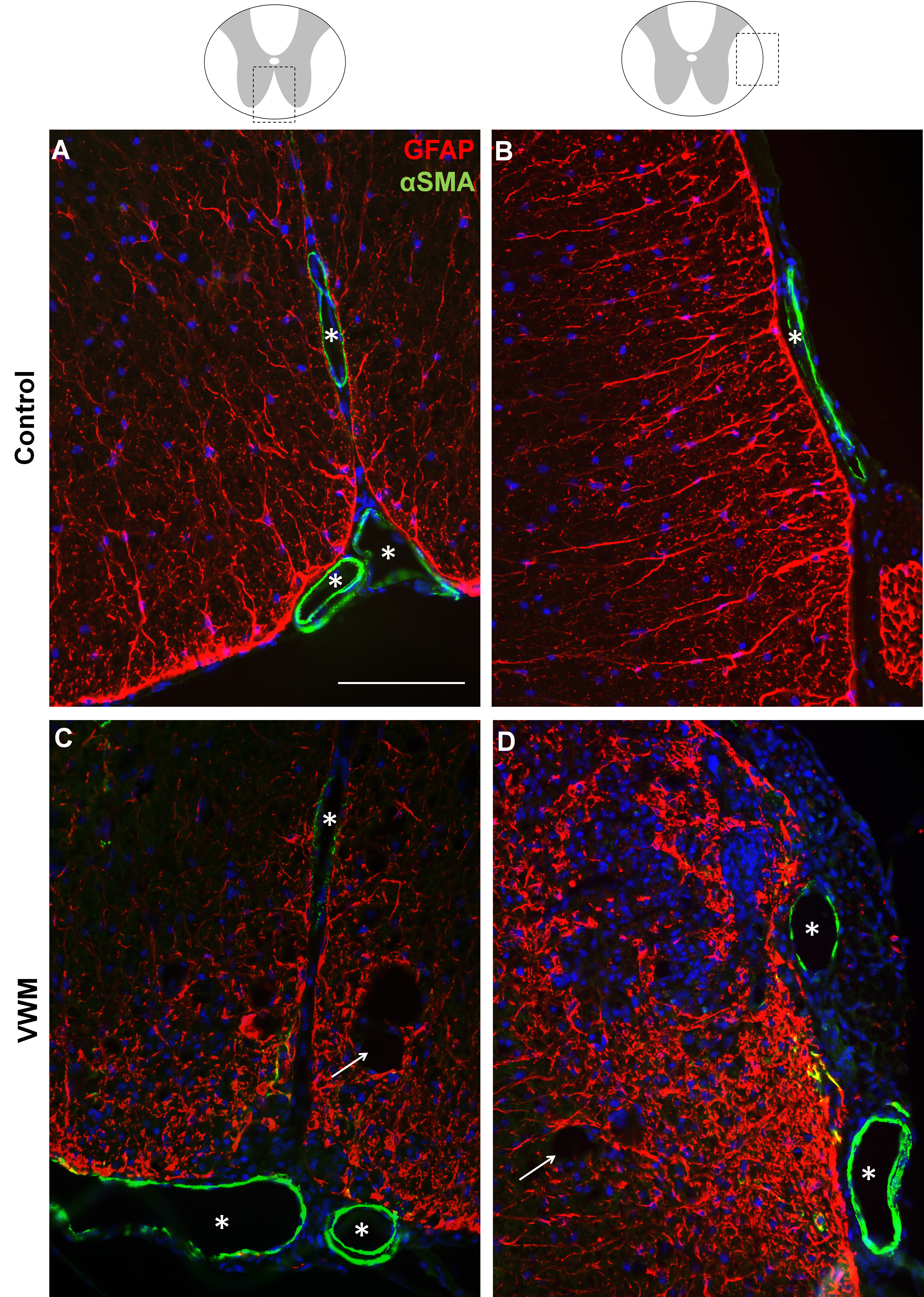

Supplement: Supplementary file 2 — Supplementary Figure 2 [file GLIA-66-862-s002.tif]

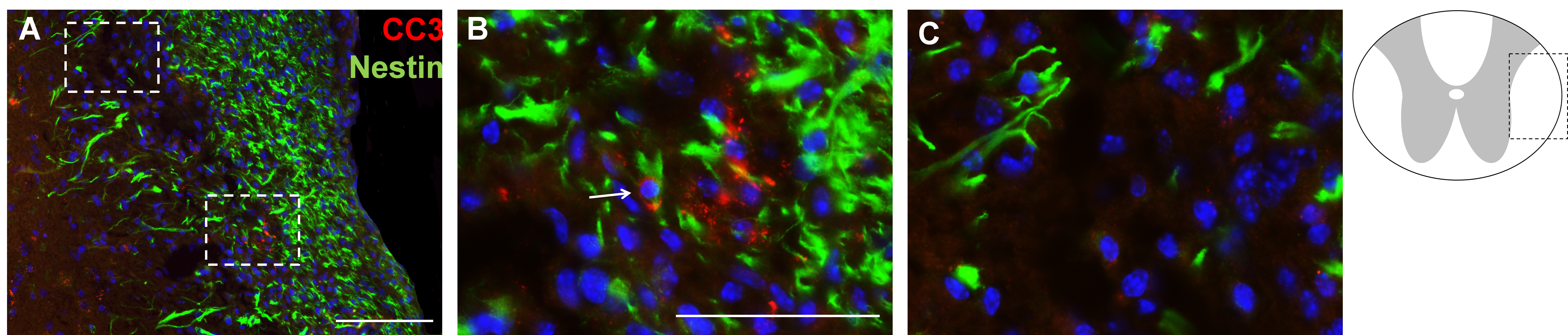

Supplement: Supplementary file 3 — Supplementary Figure 3 [file GLIA-66-862-s003.tif]

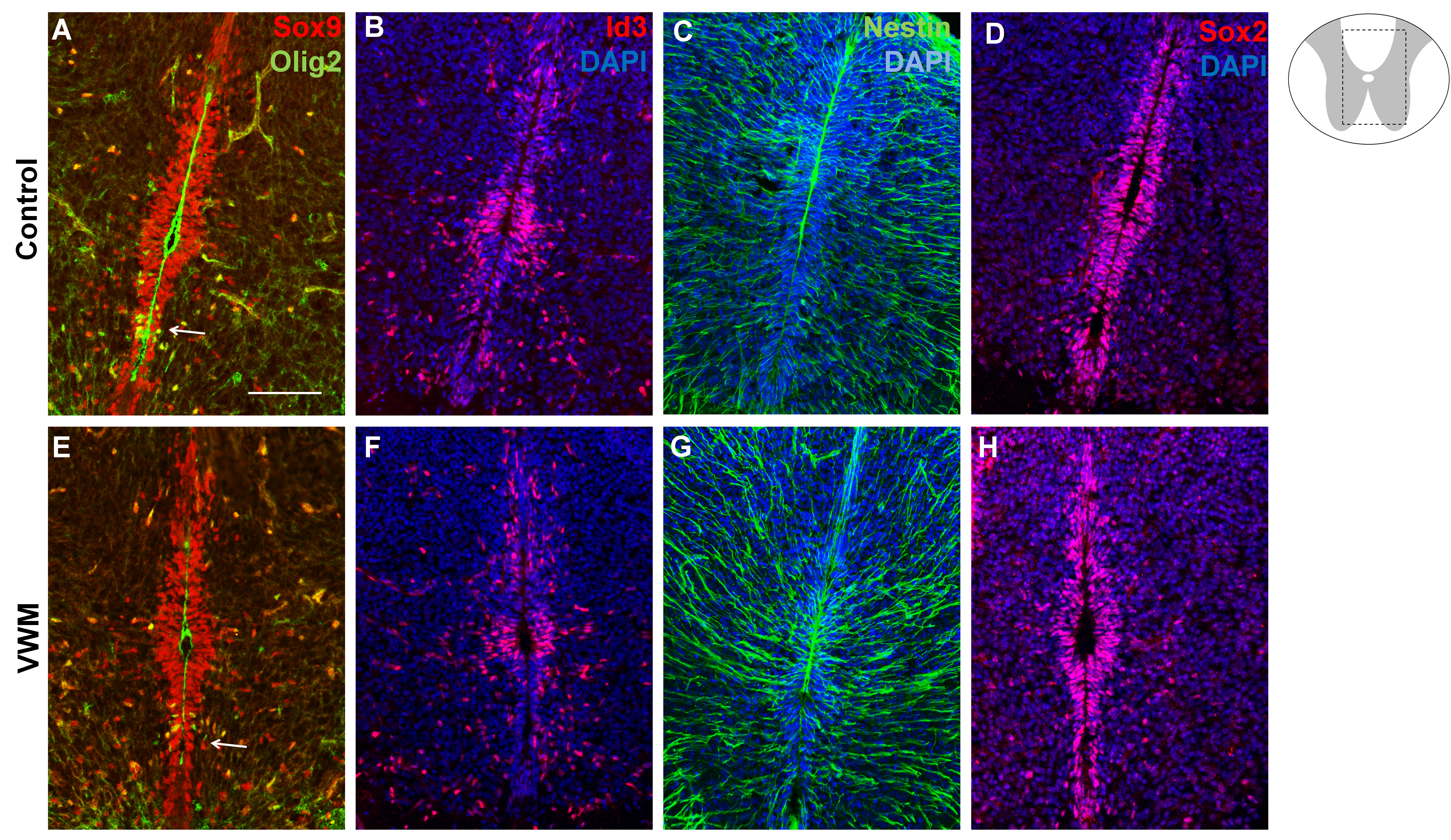

Supplement: Supplementary file 4 — Supplementary Figure 4 [file GLIA-66-862-s004.tif]
