# Supplementary material for: Affected astrocytes in the spinal cord of the leukodystrophy vanishing white matter
Source: Glia. 2017 Dec 29;66(4):862–73. doi: 10.1002/glia.23289 (PMC5838785; doi:10.1002/glia.23289)
Supplement: Supplementary file 5 — Supplementary Figure Legends [file GLIA-66-862-s005.docx]

Supplementary Figure 1. **Vimentin marks all astrocytes, not only the affected astrocyte population**. Cross sections of the lateral white matter of **A)** the cervical spinal cord of VWM patient VWM343, **B)** thoracic spinal cord of a 9 month old *2B5^he^* mouse (control), **C)** thoracic spinal cord of a 9 month old *2B5^ho^* mouse (VWM) were immunocytochemically analyzed for vimentin in combination with GFAP. Arrows indicate an example of a double positive astrocyte. Scale bar 50 μm.

Supplementary Figure 2. **Blood vessels of mouse spinal cord appear unaffected.** Cross-sections of the thoracic spinal cord ventral white matter **(A, C)** and lateral white matter **(B, D)** of the the 9 month old *2B5^he^* mouse (control, **A, B**) and 9 month old *2B5^ho^* mouse (VWM, **C, D**) were immunocytochemically analyzed for α-SMA in combination with GFAP. Asterisks indicate blood vessels, arrows indicate examples of a vacuole. Scale bar is 100 μm.

Supplementary Figure 3. **Unidentified cell masses in affected spinal cord of VWM mouse are not characterized by apoptotic cells**. **A)** Cross-sections of the thoracic spinal cord lateral white matter of the 9 month old *2B5^ho^* mouse (VWM) were immunocytochemically analyzed for CC3 in combination with nestin. CC3-expressing cells were present in the affected WM **(B)**, but most cells in the large areas with increased cell density (DAPI were negative for CC3 **(C)**. Arrow indicates an example of a CC3-expressing cell. Scale bar **A** 100 μm, scale bar **B**, C 50 μm.

Supplementary Figure 4. **Glial specification in the embryonic E13,5 VWM spinal cord is unaffected**. Immunocytochemical analysis of the E13.5 spinal cords using glial markers, **A, E)** Olig2 and Sox9, **B, F)** Id3, **C, G)** nestin, **D, H)** Sox2, in 2B5^he^ (control; **A - D**) and in *2B5^ho^* (VWM, **E - H**) mice. Sections **B – D** and **F – H** are counterstained with nuclear marker DAPI. Arrows indicate location of Olig2-expressing cells. Scale bar 100 μm.
